# 3D Bioprinted Implants for Cartilage Repair in Intervertebral Discs and Knee Menisci

**DOI:** 10.3389/fbioe.2021.754113

**Published:** 2021-10-22

**Authors:** Kalindu Perera, Ryan Ivone, Evelina Natekin, Cheryl. A. Wilga, Jie Shen, Jyothi U. Menon

**Affiliations:** ^1^ Department of Biomedical and Pharmaceutical Sciences, College of Pharmacy, University of Rhode Island, Kingston, RI, United States; ^2^ Department of Biological Sciences, University of Alaska Anchorage, Anchorage, AK, United States; ^3^ Department of Biological Sciences, University of Rhode Island, Kingston, RI, United States; ^4^ Department of Electrical, Computer and Biomedical Engineering, University of Rhode Island, Kingston, RI, United States; ^5^ Department of Chemical Engineering, University of Rhode Island, Kingston, RI, United States

**Keywords:** cartilage, bone repair, tissue engineering, 3D printing, bioprinting

## Abstract

Cartilage defects pose a significant clinical challenge as they can lead to joint pain, swelling and stiffness, which reduces mobility and function thereby significantly affecting the quality of life of patients. More than 250,000 cartilage repair surgeries are performed in the United States every year. The current gold standard is the treatment of focal cartilage defects and bone damage with nonflexible metal or plastic prosthetics. However, these prosthetics are often made from hard and stiff materials that limits mobility and flexibility, and results in leaching of metal particles into the body, degeneration of adjacent soft bone tissues and possible failure of the implant with time. As a result, the patients may require revision surgeries to replace the worn implants or adjacent vertebrae. More recently, autograft – and allograft-based repair strategies have been studied, however these too are limited by donor site morbidity and the limited availability of tissues for surgery. There has been increasing interest in the past two decades in the area of cartilage tissue engineering where methods like 3D bioprinting may be implemented to generate functional constructs using a combination of cells, growth factors (GF) and biocompatible materials. 3D bioprinting allows for the modulation of mechanical properties of the developed constructs to maintain the required flexibility following implantation while also providing the stiffness needed to support body weight. In this review, we will provide a comprehensive overview of current advances in 3D bioprinting for cartilage tissue engineering for knee menisci and intervertebral disc repair. We will also discuss promising medical-grade materials and techniques that can be used for printing, and the future outlook of this emerging field.

## Introduction

Treatment of cartilage injuries presents a significant challenge in modern orthopedics. Damage to the articular cartilage due to trauma, degenerative diseases or normal wear and tear affects everyone from children to the elderly. Poorly-healed cartilage defects cause serious and degenerative morbidities like osteoarthritis, which is the predominant cause of joint pain worldwide, affecting nearly 303 million people globally (Wei et al., 2021). The limited self-repair capabilities of the cartilage due to absence of vascularization, in which nutrients only reach chondrocytes *via* diffusion from the surrounding environment, and low chondrocyte density (comprising 1–5% of total cartilage) attributed to the high matrix to cell volume ratio, and the lack of operative and medical therapies that can significantly facilitate the healing process has led to an urgent need for new and durable treatments and grafting strategies to regenerate and repair these defects (Zhang et al., 2009; Akkiraju and Nohe, 2015; Dai and Gao, 2016).

Metal and plastic prosthetics that can recapitulate the surface of joints are currently the gold standard in cartilage and bone replacement. However, these are often rigid, limiting patient flexibility and mobility. Friction can lead to rapid wear of these implants, leading to damage and inflammation in adjacent tissues. Furthermore, the leaching of metal particles from the implants into the body can lead to adverse effects including tissue damage and poisoning (Prezyna, 2000; Sansone, 2013). Although current strategies to repair cartilage defects exist, including microfracture surgery, autologous chondrocyte implantation and osteochondral transplantation, these procedures have drawbacks such as high failure rates (25–50% within 10 years) and reduced effectiveness among elderly patients (Lansdown et al., 2018). Furthermore, autologous chondrocyte transplantation is marred by shortage of chondrocyte sources, while microfracture surgery is limited by the development of fibrocartilage instead of natural hyaline cartilage in the region, which can worsen joint function (Zhang et al., 2019; Yang et al., 2020a; Wei et al., 2021). Other methods such as debridement and spongialization have been hindered by their clinical invasiveness and the high inherent risk of developing osteoarthritis (Zhang et al., 2019).

As an alternative to existing strategies, cartilage tissue engineering is being increasingly explored as a method of fabricating functional constructs that can facilitate the regeneration of joint cartilage. Among the many strategies under consideration, 3D bioprinting has emerged as a promising method that allows precise control over the properties of the construct including shape, architecture, mechanical strength and placement of cells and bioactive cues, to mimic native cartilage. In this review, we will explore in detail current technologies being developed for the repair of joint cartilage as well as 3D printing strategies and materials being used to improve the properties and functionality of 3D bioprinted constructs for the development of functional and durable implants. In particular, we will focus on high-risk, high-load bearing and motion-critical cartilage: namely, intervertebral discs and knee menisci, for which current treatment options (e.g., implants) do not offer satisfactory biological functionality.

## Biomechanics of Joint Cartilage

In the human body, the three main types of cartilage produced include hyaline cartilage, elastic cartilage, and fibrocartilage. Hyaline cartilage, the most common cartilage found in the body, functions to provide lubrication and load-bearing support for the articulating surfaces of bones in synovial joints, essential for joint movement, in addition to playing a key role in skeletal growth and development (Krishnan, 2018). Hyaline cartilage is comprised primarily of different types of collagen, as well as proteoglycans such as aggrecan, which provides compressive strength and load-bearing support for tissues due to intermolecular repulsive interactions (Ng et al., 2003). Similar to hyaline cartilage, elastic cartilage is comprised mainly of collagen (type II) and proteoglycans. However elastic cartilage, which can be found in the ear and larynx also contains elastin fibers, allowing for increased flexibility while maintaining structural support (Chizhik et al., 2010). Knee menisci and intervertebral discs (IVDs) are comprised predominately of fibrocartilage, which exhibits high tensile strength due to the presence of thick, bundled, and highly ordered collagen (type I) fibers (Murphy et al., 2015).

### Knee Menisci

**FIGURE 1:**
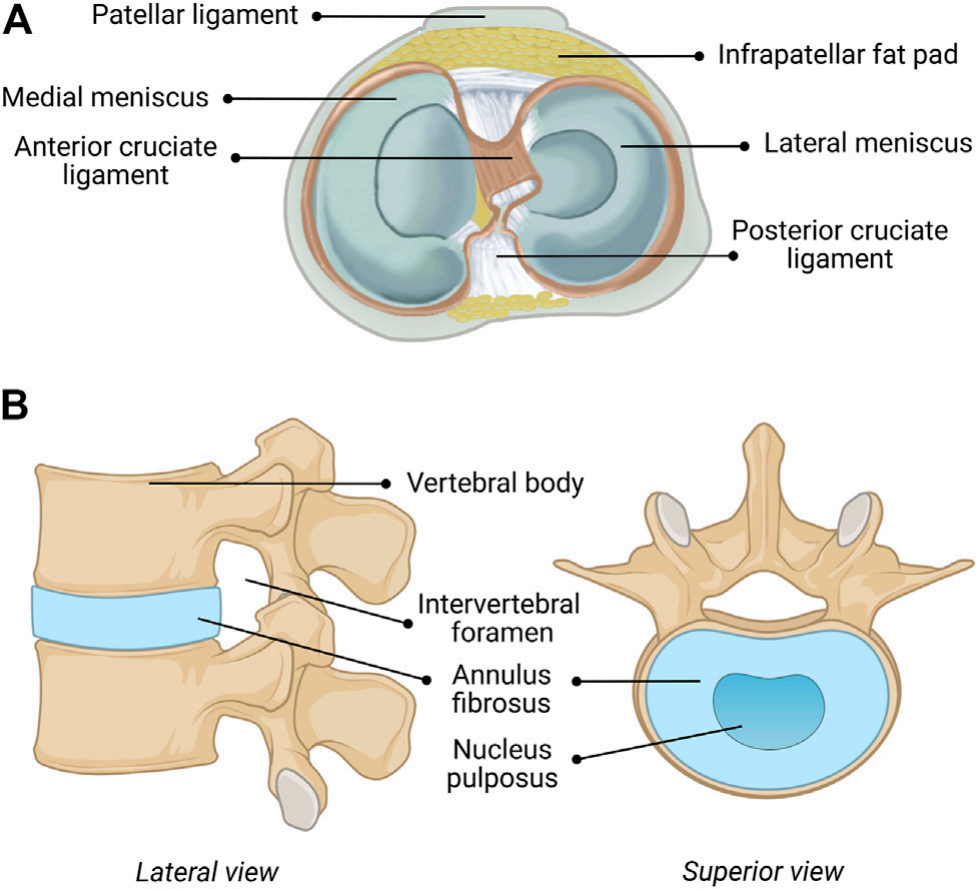
Anatomical composition of the **(A)** human knee (including menisci) and **(B)** human intervertebral disc (IVD). Figure 1A reprinted with minor alterations from (Guo et al., 2015) under Creative Commons Public Domain Dedication waiver (creativecommons.org/publicdomain/zero/1.0).

The menisci are critical for maintaining the health and performance of the knee joint, providing nutrition, lubrication, shock absorption, load distribution, and stability of the joint (Fox et al., 2012; Kester et al., 2021). The semicircular medial- and circular lateral menisci are fibrocartilaginous wedges that are attached to the femur *via* multiple ligaments, as shown in Figure 1B. The menisci are concave at the femoral surface for articulation with the condyles and flat on the tibial surface to connect with the plateau. The outer border is thick and vascular allowing for firm attachment to the joint capsule, while the inner border is thin and avascular, allowing for correct orientation within the joint. The fibrochondrocytes (meniscal cells) maintain the dense extracellular matrix (ECM) and synthesize the collagen, proteoglycans, and other proteins embedded within (Makris et al., 2011). Tissue fluid comprises 65–70% of the total weight of menisci as most of the water is sequestered within proteoglycans (Herwig et al., 1984). The orientation of the collagen bundles in the center third region are primarily radially oriented suggesting that they function in compression, while those in the outer two-thirds are circumferentially oriented suggesting a tensile function (McDermott et al., 2008). In contrast, the collagen bundles are randomly oriented in the surface layer suggesting they function to decrease friction (McDermott et al., 2008).

Material properties of the shaped human meniscus is challenging due to the complexity of the structure. Three regions (anterior, middle, posterior) of fresh-frozen human meniscus were tested in compression at four strain rates (3, 6, 9, and 12%) at a physiologically relevant walking strain rate of 32%^−s^ (Chia and Hull, 2008). Young’s Modulus increased with greater strain and was higher in the anterior region (1,048 kPa at 12%) than the posterior region (329 kPa at 12% strain), which is 8x larger than at physiological equilibrium (Chia and Hull, 2008). Stiffness in the circumferential direction is greater than that in the axial and radial direction, which are similar, while thickness varies inversely with modulus (Lechner et al., 2000; Chia and Hull, 2008; Norberg et al., 2021). Meniscal biomechanics show that the lateral meniscus demonstrates greater mobility than the medial meniscus as the lateral meniscus has 9–11 mm of anteroposterior displacement and 11.2 mm of mediolateral displacement, while the medial meniscus exhibits only 2–3 mm of anteroposterior displacement and 5.1 mm of mediolateral displacement (Thompson et al., 1991).

### Intervertebral Discs

The repair and regeneration of the cartilage of the human spine, which functions as the body’s central support system and key to movement, has been the subject of intensive research and the results have been extensively described and summarized (Panjabi and White, 1980; Oxland, 2016). The human IVD functions to give a bipedal upright human spine motion, stability, and durability. The human IVD, shown in Figure 1A, consists of three components, including a multilayered outer ring of elastic collagen tissue fibers [annulus fibrosus (AF)], which is oriented at alternating angles (50–60 + degrees) that provides stability in torsion, compression and tension (Panjabi and White, 1980; Oxland, 2016). The concentric rings do not fully form complete circles in the posterior and posterolateral regions, which make these areas susceptible to herniation, fissures and failure (Panjabi and White, 1980; Oxland, 2016). The gelatinous elastic center (nucleus pulposus (NuP)) transmits stress and weight between vertebrae (Panjabi and White, 1980; Oxland, 2016). The NuP has semi-solid-like properties and therefore expands outward when compressed, which also expands the elastic fibers of the AF. This structure is connected to adjacent vertebrae by the Sharpey’s fibers of the relatively flat cartilaginous end plates (CEP) of the vertebral bodies (Panjabi and White, 1980; Oxland, 2016). These three components, the AF, the NuP and the CEP are intimately integrated to function as a unit with the vertebrae above and below forming a spinal unit or motion unit that allows combinations of flexion, extension, lateral bending, and rotation of the spine at varying degrees depending on the location in the spine.

Mechanical properties of the human IVD are the topic of interest in many studies, and for good reason, given the debilitating results of their malfunction/degeneration (Markolf, 1974; Yoganandan et al., 1989; Iida et al., 2002; Stemper, 2010). While it had been believed that the NuP is mainly responsible for the elastic properties of the IVD, all components contribute significantly (Markolf, 1974). The compressive stiffness (E modulus: 19.5 ± 4.1 MPa in 28 ± 8 year old persons and 10.6 ± 3.4 MPa in 70 ± 7 year old persons) of the healthy IVD is 6–7 times higher than the tensile stiffness (E modulus: 2.9 ± 0.8 MPa in 28 ± 8 year old persons and 1.7 ± 1 MPa in 70 ± 7 year old persons 3.3 ± 2.1 MPa) (Iida et al., 2002; Stemper, 2010). Stiffness of the spine can vary depending on the spinal region (Shea et al., 1991). The loading of the IVD measured during normal activities was 0.1–0.5 MPa and increased up to 2.3 MPa during lifting 20 kg weight (Wilke et al., 1999). Studies on sheep IVDs showed increased stiffness of IVD after cycled loading, which fully recovered after a period of unloading (Johannessen et al., 2004).

## Current Technologies for Joint Cartilage Repair

Damage to- or degeneration of joint cartilage can lead to the development of debilitating arthritis, thereby impairing joint function (Abrams et al., 2013). In response to this, the number of research papers published on cartilage repair have nearly doubled in recent years, with the main focus being on replacement or regeneration of the knee meniscus and replacement of intervertebral discs (Abrams et al., 2013).

### Knee Cartilage

The critical role of the meniscus in knee biomechanics and joint health is well known (Kester et al., 2021). Preservation of knee meniscus by repair, allograft transplantation and partial meniscectomy in the US is the current standard of care (Abrams et al., 2013; Kester et al., 2021). Over a 7 year period, the number of meniscectomies increased by 14% while the number of meniscus repairs increased by 100% (Abrams et al., 2013). Failure rates for meniscal repair were relatively low: 12% up to 1 year, 15% up to 2–3 years, and 16.5–19% up to 4–6 years post-repair (Ow, 2021). Meniscal repairs typically result in more revision surgeries, but remain a more effective long-term treatment than meniscectomies. Accordingly, while total meniscectomy can provide short-term relief and improve function, there remains a high risk of developing osteoarthritis over the long term (Abrams et al., 2013; Kester et al., 2021; Veronesi et al., 2021). The chondroprotective effects as well as the stabilizing and load-distributing function of the meniscus are thought to have led to the increase in the choice of repair over removal (Abrams et al., 2013). Advances in arthroscopic techniques, instrumentation, and postoperative care have also likely contributed to the rise in repairs (Abrams et al., 2013).

Allograft transplants and implants show promise for restoring meniscal function. Transplant of meniscal allograft tissue to replace removed meniscus has shown encouraging results, however the availability of such tissue is limited, and can be subject to strict regulation in some countries (Veronesi et al., 2021). Meniscal scaffolds can stimulate repair and even regeneration of meniscal tissue, and is therefore an area of increasing research interest (Veronesi et al., 2021). Two acellular scaffolds are currently in clinical use, including Actifit (polyurethane-based) and collagen meniscus implant (CMI) (collagen-based). Both have demonstrated promising results in terms of moderate-to significant pain relief (measured on multiple scales) and improved movement/joint functionality over the mid-to long term (study durations lasting <10 years post-implantation) although regeneration is limited (Veronesi et al., 2021). These scaffolds remained biocompatible, while promoting limited meniscal healing, and therefore chondroprotection, which in turn decreased patient pain (Veronesi et al., 2021). Despite these early achievements, there has been a distinct lack of new meniscal implants (of any kind) approved for clinical use over the last decade.

### Intervertebral Discs

Total disc replacement (TDR) and fusion of the spinal column have been used to treat degenerative disc disease for several years (Punt et al., 2008). Despite rising rates of fusion, procedures still come with concerns regarding failure to achieve a solid fusion mass (pseudarthrosis) and adjacent segment degeneration (Salzmann et al., 2017). TDR implantation rates have remained steady in the U.S. over the last decade, possibly due to issues with correct sizing and placement of the implant, the difficult nature of the surgery, lack of device selection, or fear of postoperative complications (Punt et al., 2008; Salzmann et al., 2017). The only TDR implants currently approved by the Food and Drug Administration (FDA) are metal based (U.S. Food and Drug Administration, 2020). THE PRODISC L TOTAL DISC REPLACEMENT -P050010/S020; U.S Food and Drug Administration (2015) activL® Artificial DiscPatient Information -P120024. A006; Geisler, 2006).

Damaged IVDs of the spine can be fixed by replacing the disc with non-flexible material and then fusing the adjacent vertebrae using titanium plates, which results in reduced joint mobility (Rajaee et al., 2012). The number of patients having spinal fusion surgery increased from 203,053 to 442,776 annually from 1998 to 2014 (Sheikh et al., 2020). Despite providing temporary relief, adjacent spinal discs are often damaged due to increased stresses imposed on them as a result of lack of flexibility of fused vertebrae (Rajaee et al., 2012).

Artificial disc replacement has recently emerged as an alternative to fusion due to safer surgical procedures and better preservation of joint mobility (Park, 2015; Salzmann et al., 2017). However, total disc replacement (TDR) rates for intervertebral discs are low due to strict regulations for implant surgeries, demanding surgical techniques, low implant selection, and complications, requiring further surgery (Salzmann et al., 2017). Current TDR devices are composed of metal alloy plates sandwiching a plastic core, or a titanium mesh cage for bone infiltration that replaces the intervertebral disc, both of which can leach metal particles into the body and cause degeneration of relatively softer adjacent vertebrae and facets (FDA clears “first ever” 3D printed spine implant to treat of multiple injuries, 2018; Salzmann et al., 2017). One solution to preventing the development of adjacent segment disease is to preserve native biomechanics by replacing fusion techniques with motion sparing artificial discs. A new artificial cervical (SECURE-C TDR) metal disc has recently been developed to maintain physiologic motion, thereby reducing the risk of adjacent segment degeneration (McConnll, 2016).

**FIGURE 2:**
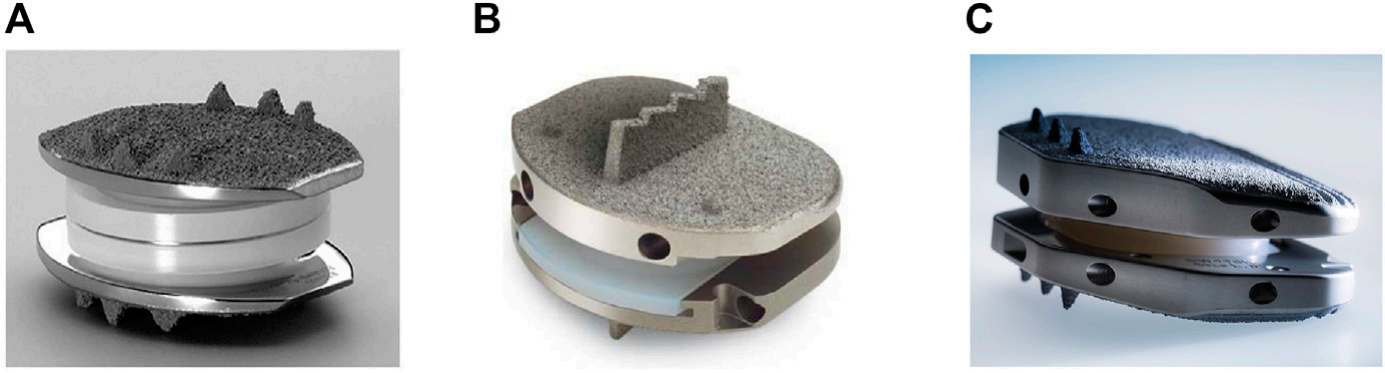
Three intervertebral disc implants currently approved for use by the FDA including: **(A)** Charité III disc replacement (Depuy Spine) and **(B)** Prodisc-L (Centinel Spine) and **(C)** ActivL^®^ artificial disc (Aesculap Implant Systems). Figures 2A,B reprinted with no alterations from (Kaner and Ozer, 2013) under Creative Commons Public Domain Dedication waiver (creativecommons.org/publicdomain/zero/1.0). Figure 2C (Aesculap’s activL® Artificial Disc product image) used with permission from Aesculap Implant Systems, LLC, Center Valley, PA.

There are currently three implants approved by the FDA for total disc replacement in the spine, including the Charité III Artificial Disc (DePuy Spine Inc., Raynham, MA), ProDisc-L (Synthes Spine, Paoli, PA) and the activL Artificial Disc (Aesculap Implant Systems, LLC), which are shown in Figures 2A,B, respectively (Salzmann et al., 2017). The Charité Artificial Disc consists of an ultra-high molecular weight polyethylene core that slides between the metallic alloy (containing cobalt, chromium, and molybdenum) endplates, with anchoring teeth on both top and bottom for attachment (Putzier et al., 2006). ProDisc-L consists of two metallic (containing cobalt and chromium) endplates and a plastic (ultra-high molecular weight polyethylene) ball-and-socket style center, with corrugated metal teeth on the top and bottom plates for attachment (U.S. Food and Drug Administration, 2020). THE PRODISC L TOTAL DISC REPLACEMENT-P050010/S020). The activL Artificial Disc is made of two cobalt-chromium alloy endplates and one polyethylene inlay. The plastic center is attached to the bottom endplate, and the top plate is designed to move over the center, allowing motion in all directions (U.S Food and Drug Administration, 2015) activL® Artificial DiscPatient Information-P120024. A006; Miller et al., 2016). There are three anchoring spikes on each of the top and bottom end plates. Additional materials used in intervertebral disc implants may include other cobalt-chromium alloys, stainless steel, titanium alloys, polyurethanes, and titanium alloy-ceramic composites (Pham et al., 2015).

Despite the successful use of metallic implants, there are still biomechanical-and toxicity concerns regarding wear debris (Salzmann et al., 2017). Wear reduces the lifetime of a prosthetic and leaves potentially harmful debris, potentially requiring the need for additional surgeries (Shankar and Kesavan, 2016). Several researchers have reported elevated levels of metal ions in the blood and urine of patients with metal-on-metal devices (Jacobs et al., 1996; Massè et al., 2003; Savarino et al., 2003). Postmortem studies have also found significant metal ion bioaccumulation in the liver, kidney, spleen, heart, and lymphatics of those patients outfitted with metallic implants (Urban et al., 2000). This metallosis is suspected to be an underreported or underdiagnosed issue, and has been shown to result in chronic inflammation, causing a host of unpleasant symptoms (nausea, cognitive impairment, hematological aberrations etc.) and more serious complications such as osteolysis or pseudotumors (Vaz et al., 2019). Further, more systemic and long-term impacts of circulating or accumulated metal ions is not completely understood, making their avoidance desirable. The AcroFlex lumbar disc replacement is one example of a metallic implant that was discontinued after poor clinical outcomes (mechanical failures, osteolysis, etc.) (Germain and Tumialan, 2012; Meir et al., 2013). There is a need to fill this gap in available synthetic and non-metallic implants that exhibit some degree of flexibility and can conserve motion while providing sufficient load-bearing support.

### Overcoming Wear: The Evolution of Traditional Cartilage Repair

Some attempts have been made to improve upon the wear-resistance of materials used for the repair of cartilage injury. These encompass various nanocomposites, metal-on-polymer arrangements, ceramics and their composites, as well as woven materials, all with the goal of increasing wear resistance and decreasing wear debris and their physiological effects. One such example is oxidized zirconium (oxinium, possessing ceramic-like properties) with cross-linked polyethylene. Originally utilized for hip replacements, this technology has recently found its way to knee replacements, wherein the ends of the femur and tibia are capped with oxinium, with a polyethylene-based disc acting as the meniscus (Bhandari et al., 2012). The expected end-result is less metal-on-metal friction and wear. While touted as a replacement to older cobalt-containing setups, long-term studies have shown almost comparable (low) rates of wear, and no reduction in revision rates (Kim et al., 2012; Zou et al., 2020). Polymer through-wear followed by wear debris release has also been documented in some patients (Frye et al., 2021). Moreover, this technology involves surgery of even greater invasiveness owing to the complicated capping process. Oxinium debris has, however, shown lower inflammation elicitation than older cobalt counterparts (Rose et al., 2012). Other ceramics have also been used in this area, although mostly for hip arthroplasty. Ceramics have a low coefficient of friction and excellent wear resistance but are generally poor in terms of fracture-resistance owing to their brittleness, and sub-optimal load-bearing and flexibility capabilities (Yup Lee and Kim, 2010). Frequent squeaking of the joints during movement is a common patient complaint when using ceramic-on-ceramic joint replacements (Jarrett et al., 2009). A quite recent development has been the exploration of woven materials: being made of polymeric or hybrid polymer-natural fiber composites, these would have less harmful wear debris. Rodts et al. presented some interesting early work in this area, using laser welding to impart high wear-resistance to their constructs, but–focusing entirely on wear resistance–did not provide any notable data with regard to mechanical properties or *in vivo* performance (Rodts et al., 2019). With no load-bearing substructure, it is difficult to envision these being applied successfully in cartilage or other constructs by themselves.

## 3D Printing for Cartilage Engineering

As a result of the limitations of current treatment options, there has been increased attention focused on cartilage and tissue engineering to overcome these limitations and facilitate joint regeneration. 3D printing is a popular type of additive manufacturing (AM) technology in which constructs are built in a layer-by-layer fashion, allowing for the design and production of patient-centric implants. 3D printing technology can be used to produce patient-personalized constructs (e.g., IVD’s) in a time- and cost-effective manner, allowing for greater flexibility in terms of material selection, typically resulting in the production of more biocompatible constructs compared to metallic implants produced *via* traditional manufacturing methods (Lim et al., 2019). Various computer aided design (CAD) programs can be used to construct digital models, typically based on patient centric data obtained *via* computer tomography (CT) and magnetic resonance imaging (MRI) scans. These models can then be imported into software capable of editing and segmenting files, retaining only particular regions of interest. Lastly, files can be uploaded into a slicing software, where printing parameters such as number of layers and layer height are determined. This process can greatly improve the accuracy and translational potential of printed constructs.

Bioprinting is a subtype of 3D printing, in which the materials used in the 3D printing process contain cells and other biomaterials to produce a final construct. It is an emerging manufacturing technique used to develop tissue engineered constructs with very precise size and shape attributes, while maintaining excellent cell adhesion and proliferation abilities. Conventionally used metallic implants for meniscus and IVD repair fail to recapitulate the complex biological environment and mechanical structure of native tissue, while also presenting potential toxicity concerns via metal particle leaching in the body (Tavakoli et al., 2020). Overall biocompatibility of printed implants is intimately linked to the materials used in the printing process, which has been the focus of many review papers in recent years (Tappa and Jammalamadaka, 2018). In addition, traditional cartilage tissue engineering approaches result in scaffolds with a homogeneous distribution of chondrocytes or cartilage progenitor cells, and fail to recapitulate the complexity of native cartilage tissue (Daly and Kelly, 2019). Through meticulous material and printing parameter optimization, bioprinting allows for the fabrication of printed constructs demonstrating precise spatial and temporal control on the placement of cells and other bioactive substances (such as growth factors) while exhibiting similar chemical and mechanical properties as native tissue to better guide tissue formation, ideal for cartilage and tissue regeneration applications (Buj-Corral et al., 2018).

To produce 3D printed implants for cartilage repair, available 3D bioprinting techniques include inkjet bioprinting, extrusion-based bioprinting, vat polymerization (VP), and laser assisted bioprinting (LAB). Currently, inkjet and extrusion-based printers remain the most common types of printers used for cartilage tissue engineering applications.

### Inkjet Bioprinting

**FIGURE 3:**
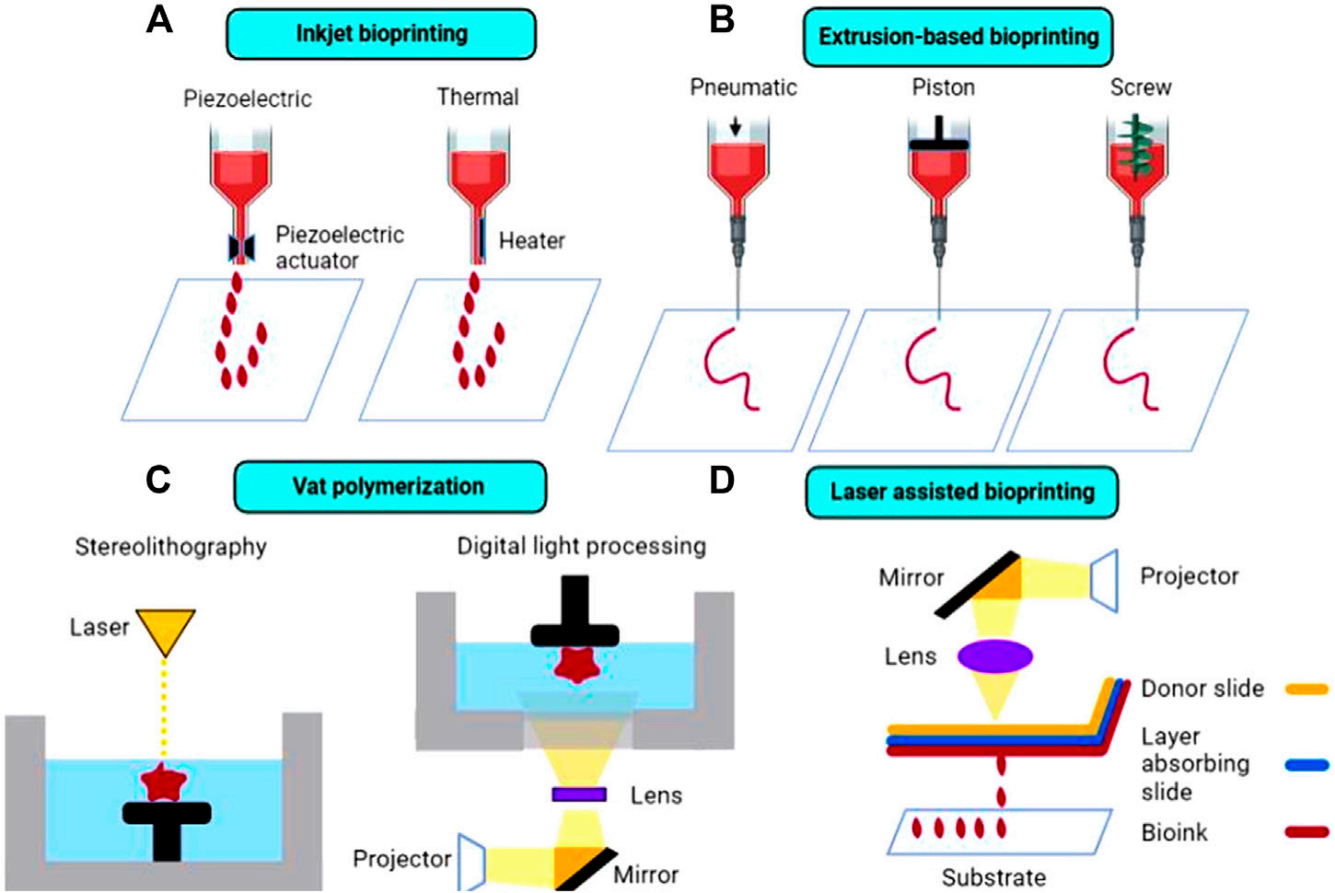
Schematic diagram of the most common 3D bioprinting techniques in cartilage tissue engineering, including **(A)** inkjet bioprinting, **(B)** extrusion-based bioprinting, **(C)** vat polymerization, and **(D)** laser-assisted bioprinting.

Inkjet printing is a type of powder bed printing technology in which binder droplets, containing cells and other biomaterials (such as growth factors) are dispensed through overhead printheads and deposited onto a substrate, as shown in Figure 3A (Shirazi et al., 2015). Inkjet printing is advantageous compared to other traditional bioprinting techniques due to the capability to produce constructs in a cost-effective and high throughput manner (Li et al., 2020). For example, Daly et al. used an inkjet printing approach to produce stratified cartilage tissue by developing a bioink consisting of mesenchymal stem cells (MSC), chondrocytes and either pluronic or gelatin methacrylate (GelMA) and injecting this bioink into polymeric microchambers to guide cell growth and more representatively mimic native cartilage tissue (Daly and Kelly, 2019). However, the droplet ejection process, which can be achieved either *via* thermal or piezoelectric actuation, can be detrimental to maintaining adequate cell viability in printed constructs due to incurred shear and thermal stresses leading to cell death (Li et al., 2016). In addition, depending on bioink viscosity, nozzle clogging can occur, preventing a smooth print from being achieved. Cui et al. (2014) modified an HP Deskjet 500 thermal inkjet printer, allowing it to print a photopolymerizable bioink containing human chondrocytes suspended in poly (ethylene glycol) diacrylate (PEGDA), suitable for cartilage tissue engineering applications.

### Extrusion-Based Bioprinting

Traditionally, extrusion-based 3D printing, including fused deposition modeling (FDM) is accomplished by feeding a solid, thermoplastic filament through a high temperature nozzle, in which the material is then continuously extruded and deposited onto a lower temperature build plate, facilitating material solidification (Long et al., 2016). Despite allowing for rapid and efficient prototyping, FDM is not suitable for thermolabile components, including cell and proteins which can degrade under high temperatures. In addition, this method is only suitable for printing using solid filaments and is not capable of printing liquid cell suspensions. Thus, commercial bioprinters have been developed in recent years to overcome these obstacles. Extrusion-based commercial bioprinters rely on either pneumatic, piston, or screw-driven configurations to achieve continuous bioink extrusion, as shown in Figure 3B. In pneumatic-based extrusion, air pressure provides the main driving force for bioink dispensing, whereas in piston and screw-driven extrusion, vertical rotational forces are exerted on the bioink resulting in extrusion (Derakhshanfar et al., 2018). It is important to note that certain configurations, such as screw-driven extrusion, may negatively impact cell viability due to large pressure drops that occur along the nozzle (Ozbolat and Hospodiuk, 2016). Commercial bioprinters are typically considerably more expensive than traditional 3D printers, which may present a significant barrier to entry for some researchers interested in bioprinting, although more cost-effective options have become available. Thus, some researchers have modified existing extrusion-based printers, enabling them to print liquid bioinks. For example, Gantumur et al. (2020) developed an alginate-based bioink containing fibroblast cells that can be printed at room temperature using a modified Prusi i3 3D printer *via* an enzyme-mediated hydrogelation method. Cell viability was similar in printed and non-printed hydrogels (54.1% in printed vs 50.4% in non-printed after 1 day of culture), showing that this printing process did not adversely impact the fate of the cells and demonstrating the feasibility of this technique for bioprinting applications.

### Vat Polymerization Bioprinting

Unlike extrusion-based printing, VP techniques rely on the layer-by-layer solidification of liquid photopolymerizable resin, containing photopolymerizable monomer(s) and photoinitiator(s), as shown in Figure 3C. A build plate moves along the z-axis inside a resin-containing vat. Upon exposure to a specific wavelength of light (dependent on resin components), the resin polymerizes and solidifies (Martinez et al., 2017). Layer adherence/formation is facilitated by unreacted monomers in the previous layer polymerizing upon light exposure. Stereolithography (SLA) and digital light processing (DLP) are the two main types of VP techniques, with the former using a laser beam and the latter using UV light from a projector to cure the resin. VP printing can overcome limitations inherent to other printing techniques, such as avoiding physical stresses imposed on bioinks (e.g., extrusion-based printing), which can ultimately lead to improved cell viability while maintaining high print resolution of final constructs (Kadry et al., 2019). Grogan et al. (2020) developed scaffold-free cartilage tissue constructs *via* a Regenova bioprinter in which microspheroids, held in place by a microneedle array, eventually fuse together to form neotissues. Li et al. (2017) combined 3D scanning technology with VP-based 3D printing (modified Bio-Architect, Regenovo) to more accurately replicate bone and cartilage defects, using either an alginate- or a hyaluronic acid (HA) based photopolymerizable hydrogel platform. Despite the advantages, concerns regarding photopolymer biocompatibility remains an issue, as unreacted resin components can present cytotoxicity issues at certain concentrations (Sabnis, 2010; Choi and Cha, 2019; Nguyen et al., 2019).

### Laser Assisted Bioprinting

LAB is a technique in which a pulsed laser source (UV or near-UV wavelengths) is focused through a donor slide onto an absorbing layer, causing immediate vaporization and resulting in an area of high vapor pressure, facilitating droplet formation of the bioink layer underneath, which then deposits onto a substrate below (Figure 3D; Li et al., 2016). Printing *via* LAB circumvents issues typically encountered in traditional bioprinting processes, such as thermal and shear stresses that often lead to lowered cell viability in printed constructs. In addition, high resolution constructs and enhanced cellular organization can be achieved *via* LAB through optimization of ink droplet characteristics, such as ink viscosity and laser energy (Guillotin et al., 2013). However, LAB is a relatively time-consuming and expensive bioprinting method compared to conventional bioprinting techniques. Keriquel et al. (2017) developed a clinically feasible *in situ* forming collagen-nano hydroxyapatite (HAp) composite bioink containing mesenchymal stromal cells suitable for bone defect repair applications using a LAB technique. The LAB setup consisted of a near-infrared pulsed laser beam coupled to a scanning mirror, which focused the laser beam onto the absorbing layer (containing a thin layer of bioink), leading to droplet generation and deposition.

## Bioinks

Materials used to successfully produce bioprinted knee menisci and IVD’s need to exhibit certain characteristics, such as biocompatibility to minimize immune response in the body; similar mechanical properties as native cartilage tissue to provide support to high load bearing regions such as in meniscus repair; and capability of promoting cell adhesion and proliferation, leading to cartilage tissue regeneration (Ahmed et al., 2019). Bioink components such as transforming growth factor beta-3 (TGF-β3) and bone morphogenetic protein-6 (BMP-6), can bind to cellular receptors, leading to cell differentiation and proliferation (Roseti et al., 2018). Daly et al. (2016) investigated properties of extrusion-based printing and the *in vitro* cartilage development achievable *via* the use of common bioinks, containing MSC and TGF-β3, including agarose, alginate, GelMA and polyethylene glycol methacrylate (PEGMA, BioINK™). Results demonstrated that agarose and alginate (which lack cell binding motifs) better supported the development of hyaline-like cartilage, while GelMA and PEGMA-based bioinks (which exhibit natural cell binding motifs and can promote cell spreading) were more suitable for fibrocartilaginous tissue development, illustrating that bioink composition plays a key role in determining cell phenotype. In addition, incorporation of polycaprolactone (PCL) fibers allowed for tailored mechanical properties to make printed constructs more suitable for load-bearing applications (e.g., articular cartilage designed for joint movement) and improve their translational potential. Examples of cartilage tissue constructs fabricated *via* different bioprinting techniques with various types of bioinks are shown in Table 1.

**TABLE 1:** Examples of cartilage tissue constructs printed using different bioprinting techniques.

Type of bioprinting	Cell type	Bioink materials	Cell viability	Ref
Inkjet	Chondro-cytes	PEGDA	90%	Cui et al. (2014)
	MSC	PEGDMA	>80%	Gao et al. (2015a)
		GelMA		
Extrusion-based	CPC	Alginate	Up to 89%	Yu et al. (2013)
	Chondro-cytes	HA alginate	>85%	Antich et al. (2020)
		PLA scaffold		
	Chondro-cytes	Alginate	85–97%	Kundu, (2013)
		TGFβ		
		PCL scaffold		
Vat polymerization	Chondro-cytes	GelMA	—	Chen et al. (2019)
		Decellularized cartilage ECM		
	Chondro-cytes	GelMA	Up to 95%	Lam et al. (2019)
		HAMA		
Laser-assisted	Mesench-ymal stromal cells	Collagen	—	Keriquel et al. (2017)
		Nano-hydroxyapatite		
	MSC	Alginate	—	Gruene et al. (2010)

CPC, cartilage progenitor cells; ECM, extracellular matrix; GelMA, gelatin methacrylate; HA, hyaluronic acid; HAMA, methacrylated hyaluronic acid; MSC, mesenchymal stem cell; PEGDA, poly(ethylene glycol) diacrylate; PEGDMA, poly(ethylene glycol) dimethacrylate; PLA, polylactic acid; PCL, polycaprolactone; TGF-β, transforming growth factor-β.

### Natural Polymers

Natural polymers with excellent biocompatibility and biological activity are an ideal class (relative to synthetic polymers) of materials suitable for use in bioinks, as they are capable of supporting cell attachment and differentiation (Liu et al., 2018). Despite the inherent advantages, natural polymers typically exhibit weaker mechanical properties than synthetic polymers, which can lead to poor printability and hinder *in vivo* performance. Constructs produced using solely natural polymers are typically more prone to failure within the body, as their mechanical properties are often not sufficient to withstand forces exerted *in vivo*. Thus, addition of materials that exhibit enhanced mechanical properties to form composites, and optimization of material crosslinking are strategies that are often used to develop more suitable natural polymer-based bioinks (Peng et al., 2020).

Alginate is a polysaccharide originally derived from brown algae and is often used to produce constructs capable of mimicking ECM structure by crosslinking with calcium ions (Ca^2+^). Alginate is a promising bioink material due to its biocompatibility and rapid gelation process, leading to suitable printability. Yu et al. (2013) produced tubular channels, designed to mimic native vasculature, *via* an extrusion-based printer using a sodium alginate based bioink containing cartilage progenitor cells, capable of achieving cell viability up to 89%. To improve printability and cell proliferation ability of alginate-based bioinks, composite materials have been developed. For example, Antich et al. (2020) developed a HA-alginate composite bioink able to produce chondrocyte-laden constructs that exhibited improved mechanical properties (e.g., increased viscosity) leading to enhanced printability, while maintaining impressive cell viability (>85%) immediately after printing, with similar cell viability up to 4 weeks later.

HA is a linear polysaccharide and natural glycosaminoglycan (GAG) that plays numerous roles in the human body, such as maintaining ECM structure and acting as a signaling molecule capable of interacting with many cell surface receptors (Dicker, 2014). HA is a biocompatible and biodegradable material and can be easily chemically modified to alter its biological functions, thereby making it an ideal material for cartilage tissue engineering applications. However, like other natural polymers, HA exhibits relatively weak mechanical properties, leading to decreased printability. Ouyang et al. (2016) developed a 3D printable modified HA-based hydrogel ink, relying on guest-host supramolecular assembly *via* adamantane and β-cyclodextrin coupled to HA, resulting in increased viscosity and storage modulus values for modified HA hydrogels. In addition, fibroblast cells were seeded onto printed hydrogels and displayed adequate cell adhesion, illustrating the potential for this material to be used for cartilage tissue engineering applications. Park et al. (2014) developed a bioprinted (in-house extrusion based printer) multicompartment hydrogel platform designed to promote osteochondral tissue regeneration, comprised of a chondrocyte-encapsulated HA compartment and osteoblast-encapsulated collagen (type I) compartment, capable of maintaining good cell viability of both cell types.

Collagen is the most abundant protein in the human body, and has been used to produce bioinks that effectively mimic the ECM structure while promoting cell adhesion, proliferation, and migration (Somaiah et al., 2015). As with other natural polymers, collagen suffers from relatively weak mechanical properties. Rhee et al. (2016) developed a bioink consisting of high concentration (>10 mg/ml) pure collagen containing meniscal fibrochondrocytes, and demonstrated that fabricated constructs could not accurately recapitulate the mechanical properties of native meniscal tissue. Shim et al. (2016) developed a multilayered construct using a bioink comprised of modified HA (Cucurbit [6]uril and 1,6-diaminohexane (DAH)-conjugated HA) and pepsin-treated collagen (atelocollagen) containing human turbinate-derived mesenchymal stromal cells as well as additional biomaterials (e.g., BMP-2 and TGF-β) capable of inducing cartilage regeneration in an *in vivo* rabbit model.

Gelatin is a linear peptide produced from the denaturation of collagen. Gelation can occur *via* chemical/enzymatic reactions or physical crosslinking, which requires heating and subsequent cooling to produce a semi-solid gel (Bello et al., 2020). Gelatin is an attractive bioink material due to its biocompatibility, biodegradability, and ease of modification (e.g., GelMA), allowing for facile crosslinking and enhanced cellular interactions (Gungor-Ozkerim et al., 2018). However, crosslinking of pure gelatin can be slow and may result in printed constructs that are mechanically inferior to native tissue. To overcome these issues, Singh et al. (2019) developed a gelatin encapsulated composite bioink containing a silk blend (*Bombyx mori* and *Philosamia ricini*) designed to enhance bioink mechanical properties, leading to enhanced printing fidelity while promoting the growth and proliferation of chondrocytes for cartilage tissues regeneration applications. Bioinks have also been developed containing modified gelatin, such as GelMA, in which the degree of methacryloylation and GelMA concentration have been shown to play a key role in modulating overall mechanical properties, including viscosity and stiffness (Leucht et al., 2020).

Other natural polymers, such as silk fibroin and elastin have recently been used in bioinks to produce more physiologically relevant IVD components (i.e., annulus fibrosus) (Costa et al., 2019). Such constructs demonstrate tunable degradation profile and mechanical properties, while remaining biocompatible. On a similar note, Tamo et al. developed a 3D printable chitosan-based hydrogel reinforced with cellulose nanofibers to better address the needs of developing mechanically demanding tissue repair strategies, for use in IVD and menisci repair applications, amongst others (Kamdem Tamo et al., 2021).

### Synthetic Polymers

Compared to natural polymers, synthetic polymers such as polycaprolactone (PCL), polyethylene glycol (PEG), and PEG derivatives, such as Pluronic, exhibit more suitable mechanical properties, and are thus better able to withstand the forces exerted during the bioprinting process (Liu and Wang, 2020). In addition, they can support the development of porous structures and microchannels, which serve to recapitulate the vasculature of native tissue. Typically, however, synthetic polymers lack the biological activity necessary for promoting cell growth and proliferation and are thus used primarily for their role in providing structural support for bioinks (Wu et al., 2011).

PCL is a slow-degrading biocompatible polyester that has been used previously in bone tissue scaffold fabrication, and other medical products such as suture and bone screws (Chen et al., 2015; Cho et al., 2019; Dwivedi et al., 2020). PCL is commonly used in bioprinting applications to enhance the mechanical properties of printed constructs. Previous studies have supported this notion, as constructs have been fabricated using PCL as a scaffold material demonstrating an increase in elastic moduli that more closely mimic native articular cartilage (Daly et al., 2016). Kundu (2013) developed a chondrocyte-containing alginate-based bioink able to be printed onto a PCL scaffold using an extrusion-based multi-head deposition system for cartilage tissue engineering. In this study, PCL was used as a support structure for the cell-laden alginate hydrogel, while facilitating cartilage tissue regeneration up to 14 days post-implantation, as demonstrated using a nude mice model.

PEG is a highly tunable, relatively inert synthetic polymer synthesized *via* the polymerization of ethylene oxide, that exhibits favorable mechanical properties suitable for cartilage tissue applications. Poloxamer, also known as pluronic, is a PEG-based block copolymer and is also commonly used in bioink development. PEG-based derivatives are attractive bioink materials due to their tunable gelation and favorable mechanical properties, both of which can be modulated by altering pluronic concentration (Geng et al., 2011). For instance, Ribeiro et al. (2018) investigated the printability of PEG/poloxamer-based bioink blends and demonstrated that bioink mechanical properties could be tuned by changing the PEG/poloxamer ratio, noticing that as this ratio increased, mechanical properties (i.e., yield stress, viscosity and storage modulus) decreased correspondingly. PEG and PEG derivatives have been combined with other biologically-active materials to facilitate cell adhesion and proliferation on printed constructs (Hutson et al., 2011; Rutz, 2015). For example, Armstrong et al. (2016) developed a pluronic-alginate composite bioink capable of producing constructs exhibiting a highly porous structure ideal for cell growth and nutrient transport, that supported the proliferation and differentiation of hMSCs over a 5-week period. In addition, photopolymerizable PEG-based materials, such as PEGMA, PEGDA and polyethylene-tetracrylate (PEGTA)–which can crosslink *via* light irradiation–have been developed and are commonly used in bioink formulations for their tunable mechanical properties and facile crosslinking abilities (Skardal et al., 2010). Gao et al. (2015b) developed a hMSC-encapsulated photopolymerizable PEGDMA-peptide bioink demonstrating good printability capable of producing scaffolds promoting bone-and cartilage regeneration.

## Cell-Based 3D Bioprinting

**FIGURE 4:**
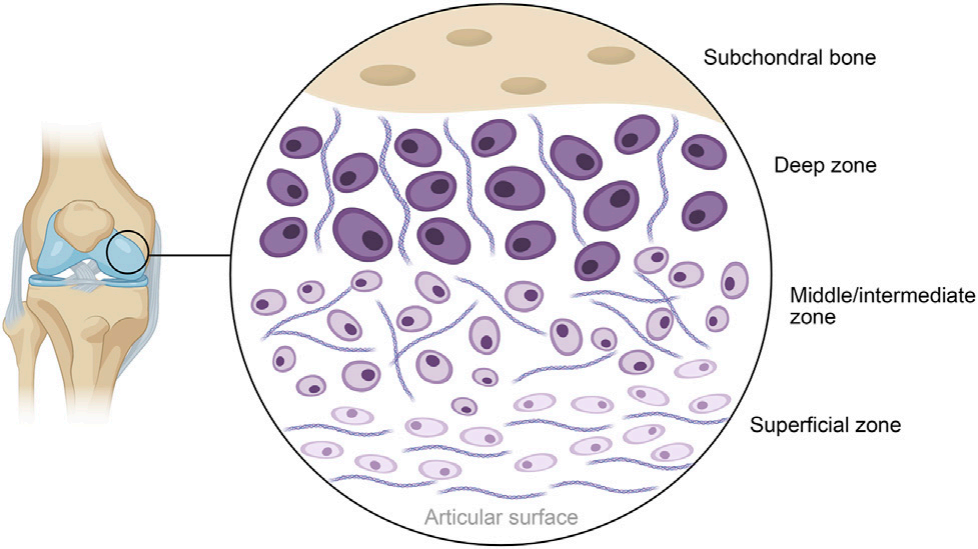
Schematic diagram of the general structure of human articular cartilage, shown in the context of the knee. The deep zone contains hypertrophic chondrocytes interspersed with radially arranged collagen fibers, progressing to polymorphic chondrocytes and randomly arranged collagen in the middle layer. The superficial layer closest to the articular surface contains flattened chondrocytes packed in between horizontally arranged fibers of collagen.

The earliest record of 3D bioprinting with cells being utilized for the regeneration or repair of cartilage is provided by the work of Cui et al. (2012). Fine spatial control and print resolution is highly useful when one considers the distinct structural zones that exist within articular cartilage, particularly the stratification by cell type. Chondrocyte organization, shape and expression patterns have been observed to vary markedly across three distinct layers: the superficial-, middle- and deep zones, as shown in Figure 4 (Akkiraju and Nohe, 2015). Alternatives to 3D bioprinting such as micromolding or other conventional techniques struggle to recreate these delicate features without significant and painstaking work, often with no guarantee of success. Bioprinting can allow cells with pre-induced expression profiles to be printed in distinct organizational patterns, densities, and depths, while other techniques with low control makes achieving local homogeneity in cell distribution a tough task. Tight spatial control also allows for the use of multiple polymer or matrix materials in complex patterns to achieve desired local-or global mechanical characteristics. This cell-friendly fabrication process allows cells to be seeded earlier on in the fabrication process, further improving fabrication times that have already been made shorter by bioprinting’s automated nature. In all, these serve collectively to produce high quality, highly biomimetic cartilage structures.

### Cell-Based Bioprinting of Knee Cartilage

Articular cartilage of the knee is one of the most commonly damaged type of cartilage in the human body, as a result of a variety of factors such as age, physical activity or diseases such as osteoarthritis, compounded by the joint’s sheer complexity and load-bearing role (Gracitelli et al., 2015). As a result, most bioprinted cartilage applications have focused on regenerating the cartilage of the knee. Given that articular cartilage damage frequently co-occurs with lesions on the subchondral bone, a recent paper by Yang et al. (2020b) sought to produce an integrated, 3D bioprinted construct capable of regenerating both the damaged cartilage and the bone below it. To this end, they printed a layered composite consisting of sodium alginate and gelatin in the “cartilage” layer, and sodium alginate, gelatin and hydroxyapatite in the “bone” layer (the latter being an essential component of the bone regeneration process) with pore sizes of approximately 500 μm (Kattimani et al., 2016). These inks were seeded with pre-differentiated osteogenic and chondrogenic bone marrow mesenchymal stem cells (BMSCs) prior to implantation on rabbit knee lesions for *in vivo* studies. The mechanical properties of this construct were inferior to that of natural cartilage, with a compressive strength of 11 MPa after 6 months post-implantation (30 MPa in natural cartilage) and a maximum tolerable load of 183 N (480 N in natural cartilage), which may be attributed to the mechanical properties of the chosen bioink in this study. Despite this, cell viability *in vitro* was sufficiently high (>70% by day 7) and mechanical properties were significantly better than cell-less controls. Notably, the complete implants could produce hyaline-like cartilage with a still-disorganized structure and delicate links to the surrounding cartilage by 3 months post-implantation, and by 6 months, was histologically indistinguishable from the surrounding cartilage, although a small ‘transition area’ was visible. This stood in contrast to blank or even cell-less controls where repair either did not happen or was much slower and did not show the key integration with the surrounding cartilage or the subchondral bone. Another research group attempted a similar approach, printing a HAp-doped gelatin hydrogel (10–50 μm pore size) with human umbilical cord blood-derived mesenchymal stem cells (Huang, 2021). The construct was demonstrated to possess a compressive modulus of approximately 77 kPa, with HAp’s role in lending compressive strength apparent in the lower compressive modulus of gelatin-only constructs (70.5 kPa). This work demonstrated that HAp was able to induce a certain degree of chondrogenic differentiation as well, noting upregulation in the expression of chondrogenic markers aggrecan (ACAN) and Collagen Type II Alpha 1 Chain (COL2A1) from day 7 on, while levels of SRY-Box Transcription Factor 9 (SOX9) were elevated considerably from day 14 on. The authors did not, however, examine effects of the HAp on the construct’s interaction with the subchondral bone, focusing instead on the HAp’s ability to induce differentiation, provide strength/stiffness and their ability to act as excellent surfaces for chondrocyte growth and proliferation. In all, this integrative approach is one that merits inclusion in more works in the field of bioprinted cartilage.

The meniscus of the knee has also garnered the attention of 3D bioprinting, mostly due to the inability of current techniques of meniscus repair to address damage deeper in its structure (Guo et al., 2021). Luo et al. (2020) described the design and optimization of a bioink of gelatin, alginate and cellulose nanofibers loaded with rabbit fibrochondrocytes (rFCs). Several different formulations were tested, with pore size increasing proportional to both gelatin and cellulose content: low-gelatin alginate modified with cellulose nanofibers had pore sizes of approximately 180 μm relative to the 300 μm observed in high-gelatin alginate modified with cellulose. Their final construct was printed based on translated MRI imaging data and was thus patient-specific; it could maintain very high cell viabilities (>90% over 14 days) and displayed markedly high levels of collagen types II and X (Col II/X) *in vitro*. These are both indicators of healthy cartilage, the latter being indicative of the hypertrophic chondrocyte phenotype found deep in cartilage structure.

**FIGURE 5:**
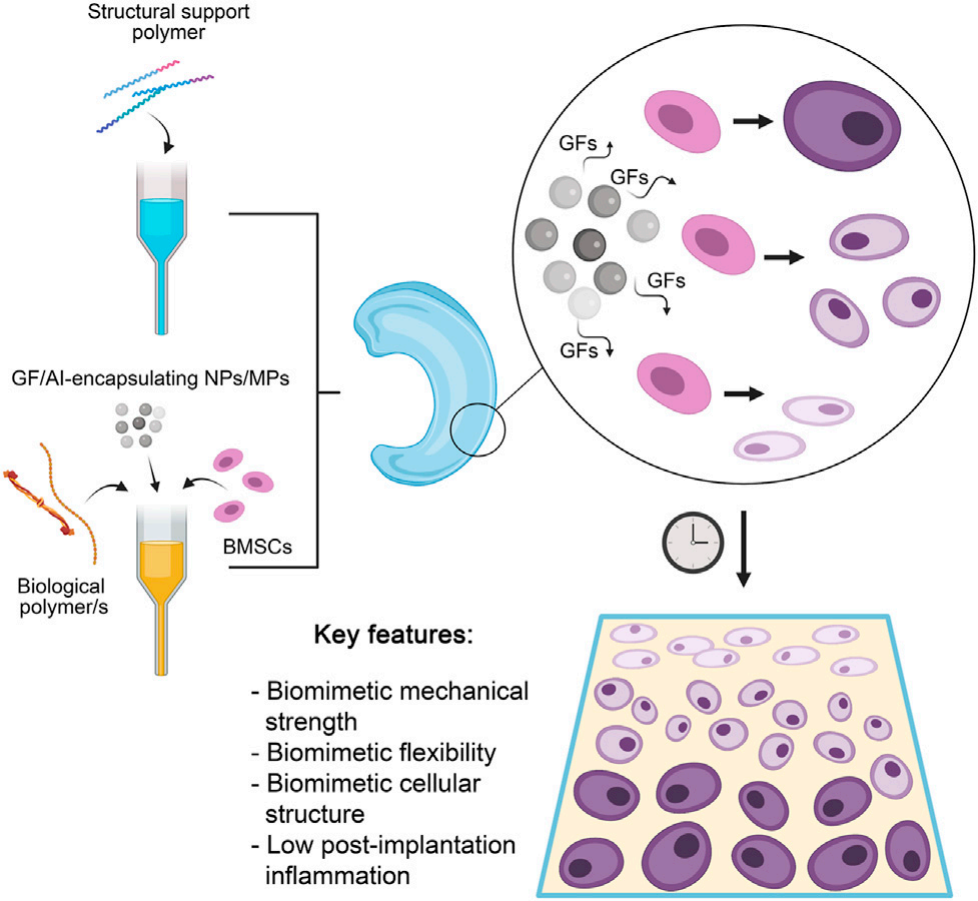
A summary of the most optimal current approach to 3D bioprinting of cartilage structures. A structural support polymer (usually synthetic) is chosen based on desired load-bearing capabilities and co-printed with a range of biological polymers that provide elasticity and a growth medium for seeded cells. Also included are stem cells capable of differentiation into chondrocytes, and nano- (NPs) or microparticles (MPs) encapsulating various growth factors (GFs) to induce differential differentiation of stem cells and anti-inflammatory agents (AIs) to reduce post-implantation inflammation. The release of GFs results in a gradual differentiation of seeded stem cells into mature chondrocytes in a stratified manner. The resultant construct has several key features that allows successful clinical translation.

The incorporation of growth factors (GFs) and other molecules to initiate *in situ* differentiation of cells in bioprinted cartilage constructs (as illustrated in Figure 5) has been a growing area of interest. Several groups have published on this topic in recent years, examining in various ways how GF can influence cell-laden 3D bioprinted constructs (Henrionnet et al., 2020; Chawla et al., 2021). This has provided an impetus for the printing of constructs pre-loaded with GFs, in a major shift towards biological biomimicry over simple mechanical or morphological biomimicry. Sun et al. (2019) have been frequent contributors to the area, with one of their earlier works translating the findings of a genomics investigation into a viable cartilage regeneration/replacement strategy using 3D bioprinting. Having identified growth differentiation factor 5 (GDF5) as a chondroprotective agent capable of inducing chondrogenic differentiation in rabbit BMSCs, they encapsulated this agent within poly lactic-co-glycolic acid (PLGA) microparticles (MPs) which were then printed into the interfibrillar spaces of a PCL-based support structure, alongside a composite hydrogel made of gelatin, fibrinogen, hyaluronic acid and glycerol laden with rabbit BMSCs. More than 80% of encapsulated GDF5 was observed to be released from the microspheres within 60 days (*in vitro*), and cell proliferation increased exponentially *in vitro* in the 21-day period tested. Most notably, ultimate tensile strength (UTS) was very close to native knee cartilage, as implants exhibited a UTS value of 24 MPa vs. the physiological UTS of 28 MPa. *In vivo*, GDF5 laden constructs were better able to produce hyaline-like neocartilage than constructs without GDF5 MPs, alongside higher GAG and Col II (also indicators of healthy native cartilage, and of chondrocyte phenotype) and displayed better cell spreading and proliferation. Continuing their work, the authors employed 3D bioprinting in the construction of a goat meniscus using a similar approach as above (Sun et al., 2021b). A hydrogel of the same type loaded with goat BMSCs and PLGA MPs containing connective tissue growth factor (CTGF) and (TGFβ3) were printed within a PCL scaffold. Of interest were the conversion of imaging data of a goat meniscus into a 3D model for the print and the differential distribution of the MPs within the meniscus (CTGF-containing MPs in the outer 1/3, TGFβ3 MPs in the inner 2/3) to stimulate differential BMSC differentiation, an improvement upon the indiscriminate differentiation in their previous work. Levels of SOX9 were observed to be elevated by 2x (CTGF MPs) to 3.5x (TGF β3 MPs) *in vitro*, relative to controls. Similar patterns of GAG and Col II vs Col I in the inner- and outer layers were also observed as in their previous work. *In vivo*, qualitative data showed that goats implanted with the complete construct had better 24-week mobility than those with control implants. Histological analysis of the implanted menisci in that period showed the expected native structure of an inner layer with an abundance of Col I-expressing fibroblast-like cells and the outer layer with Col II-expressing chondrocyte-like cells. Post-24 week implants displayed mechanical properties nearly identical to native cartilage, with statistically insignificant differences. Ultimate tensile strength, for instance, stood at approximately 29 MPa in native cartilage *vs*. 28 MPa in the implants; overall tensile modulus was higher in the implants by approximately 10 MPa. Radial and circumferential (both outer and inner layers) tensile modulus readings showed that implants lagged behind native cartilage by margins of less than 5%. By contrast, Deng et al. employed human parathyroid hormone (PH) to prevent chondrocyte hypertrophy in order to achieve and maintain hyaline-like properties (Deng, 2021). They achieved this using a biphasic, layered 3D bioprinted construct made of two distinct bioinks. The first was a mix of GelMA and PH-conjugated SF loaded with rabbit articular cartilage cells, while the second was a mix of GelMA and methacrylated silk fibroin (MSF) loaded with BMSCs, whose pore sizes ranged from 150 to 300 μm. The SF-containing construct was shown to possess a compressive elastic modulus of approximately 102.5 kPa *vs*. 211.1 kPa in the MSF-containing constructs; compressive elastic strength was also higher when MSF was present (260 kPa, a 4x increase over the SF-containing constructs)*.* Significantly lower levels of collagen X and matrix metalloproteinase 13 (MMP13) were expressed by cells grown in the complete construct relative to PH-negative controls. PH was also successful in engendering higher levels of Col II and ACAN in the cells, further confirming its role in maintaining hyaline-like phenotype. *In vivo,* the complete construct had filled lesions on rabbit knee cartilage by week 12 post-implantation. Yang et al. (2021) combined both GFs and MSC-recruiting aptamers in an interesting novel approach. Carbodiimide-mediated conjugation of HM69 aptamer was carried out on decellularized ECM, which was in turn dissolved in TGFβ3-containing GelMA. This bioink was then co-printed with PCL to produce a layered, dual-functional lattice structure with a heterogeneous macro- (800–1,000 μm) and microporous (80–200 μm) structure. The compressive modulus of this material was observed to be approximately 25 kPa, comparing favorably to expected *in vivo* physiological strain. While cells were later externally cultured on these constructs, what is of note was the ability of the HM69 to recruit adipose-derived stem cells deeper into the construct both *in vitro* and *in vivo*. These GF-loaded, bioprinted constructs are of great use to this field as cell differentiation properties of printed constructs remains paramount to the success of biomimetic cartilage replacement/regeneration strategies. In particular, the inclusion of GFs in extended release micro-/nanoformulations into 3D bioprinted cartilage constructs circumvents a key stumbling block these constructs face during clinical translation-namely, the lack of long-term GF-based support once implanted (Francis et al., 2018).

Recognizing the need for anisotropy for clinical translation, Sun et al. (2020a) have also investigated the significance of construct pore sizes on vascularization and oxidative stress (which in turn influence cellular differentiation). Their design was a four-layer 3D bioprinted construct with gradients of both pore size and GFs. Using their BMSC-loaded, four-component hydrogel within a PCL support referred to previously, the construct was made such that the first layer was endowed with a pore size of 150 μm, which steadily increased to 750 µm in the final, innermost layer. This gradient was designed to accommodate and encourage differentiation of rabbit BMSCs into the smaller flattened chondrocytes observed in the superficial zone of natural cartilage, then into the progressively larger polymorphic- and columnar chondrocytes of the middle zone, and finally, the hypertrophic chondrocytes of the deep zone. This approach was supplemented with PLGA microparticles encapsulating bone morphogenetic protein 4 (BMP4) in the innermost layer of the construct and transforming growth factor-β3 (TGFβ3) in the outermost/superficial one.

*In vitro,* a clear gradient in the expression of COL2A1 and proteoglycan 4 (PRG4) (markers of the superficial zone of natural cartilage) was observed, decreasing steadily from superficial to deep layers (12x *vs* 1x for PRG4 and 9x *vs* 1x for COL2A1). Cells in this region were observed to be fibroblast-like. Conversely, the levels of Collagen Type X Alpha 1 Chain (COL10A1; cartilage deep zone marker) were 9 times higher in the deep layer (relative to superficial), resulting in clear chondrocyte hypertrophy. The authors also managed to match physiological mechanical properties (a Young’s modulus of approx. 300 MPa 12 weeks post-implantation), maintain >70% cell viability *in vitro* and confirm good functional capabilities/performance *in vivo* (with consistently higher histological scores and virtually indistinguishable integration with surrounding cartilage by week 24, relative to single-GF controls. Better microvessel ingrowth was also observed in the dual-GF constructs). These so-called ‘dual stimuli’ constructs also fared better than controls that either only had the pore size gradient alone or the growth factor gradient alone. Yet another work by the same group directed these anisotropic 3D bioprinting efforts at improving their meniscus construct mentioned previously (Sun et al., 2020b). Seeking again for better biomimicry, they repeated their goat meniscus work within a rabbit model, this time with the addition of microparticles carrying magnesium ions for enhanced vascularization of the superficial zone and skeletal muscle stem cells in place of BMSCs. Again, gradient cell differentiation was recapitulated, and the neovascularization of the superficial layer was observed to closely match the vascular pattern observed in natural menisci (confirmed with endothelial tube-forming assays, which showed the construct was highly capable of inducing the same). *In vivo*, the constructs matched natural cartilage in morphology, histological aspects, and mechanical properties by week 24 (the latter being almost identical to those seen in their earlier work). Anisotropy is a key feature of natural systems and replicating this is especially challenging *in vitro*. Nevertheless, these works discussed above have shown how 3D bioprinting can overcome this issue, and how established growth factor-loaded systems can be adapted for such. Moreover, vascularization of tissue constructs (vital particularly for menisci, which are naturally vascularized along one facet) has long been a significant obstacle for clinical translation of these technologies (Laschke and Menger, 2012; Francis et al., 2018). The work last discussed in this section has elegantly transcended this problem, combining anisotropy, spatial control of cell/ink placement and controlled release of GFs to set a benchmark for the field.

### Cell-Based Bioprinting of Intervertebral Discs

Damage to the cartilage of the intervertebral discs (IVDs) can be particularly debilitating, given the key role the spine plays in support and movement. Recapitulating–both biologically and mechanically–the AF and NuP are important steps in producing replacements or regeneration strategies for IVDs. Composed of GAG and Col II, the NuP is a softer, elastic tissue at the core of the IVD, helping distribute pressure through it. The surrounding, harder AF is composed of collagen type I (Col I), and acts as the tougher, load-bearing and shape-maintaining portion of the IVD (Sun et al., 2021a). Bioprinting efforts in this area (particularly in mimicking the AF) have been stymied by the lack of dedicated bioinks for IVDs, as others have already pointed out (Tavakoli et al., 2020). A notable example of a novel bioink for IVDs was presented by Hu et al., who combined thermoplastics and hydrogels in a tunable, 3D printed cartilage system using gellan gum-poly (ethylene glycol) diacrylate (GG-PEGDA) and PLA (Wu et al., 2018). A dual-nozzle printing mechanism formed an outer ring of PLA to replicate the stiffer AF and GG-PEGDA hydrogels seeded with murine bone marrow mesenchymal stem cells (BMSCs) occupying the cavities of the PLA honeycomb substructure standing in for the softer NuP. A Young’s modulus of 184 kPa and a compressive strength of 55 kPa was observed in the final construct. The near-total degradation of the PLA within 6 months, excellent cell viability (>90%, 7 days) within the GG-PEGDA hydrogels and progressively high levels of F-actin observed through immunostaining after 7 days (indicating good cell spreading through the construct) were all signs that their bioprinted structure would be a highly biomimetic IVD replacement *in vivo.* Stevens et al. provides an overview of the development of GG-based bioinks, while a recent review by Pieri *et al.* provide a more comprehensive review of possible novel bioinks for IVD (Stevens et al., 2016; Pieri et al., 2020). Notwithstanding advances in this field, truly biomimetic IVD replacement/regeneration strategies are only beginning to be researched. Two recent pieces of work stand out for being on this cutting edge. Wu et al. (2021) recently outlined a PLA-based scaffold printed parallelly alongside GG-PEGDA/rat BMSCs, with the NuP and AF differentiated by the patterning of the printed strands, aiming to mimic the porosity and mechanical strength of the two. Of interest was the achievement of an excellent, physiological Young’s modulus (remaining >8 MPa over 14 days). When combined with the >80% *in vitro* cell viability 2 weeks post-seeding, the maintenance of >75% disc height *in vivo* over 6 months and comparable levels of proteoglycan expression by the bioprinted implant as in the case of reimplanted natural IVDs over the same period, this is a clear sign that properly designed and -executed thermoplastic-hydrogel composites still hold real promise in this area. The authors did not, however, examine cellular differentiation along their NuP-AF axis, crucial for true biomimicry. Sun et al. (2021a) combined biomaterials, polymers, GFs, cells, and NPs in a complex milieu to form a highly biomimetic IVD replacement capable of inducing site-specific cell differentiation over time. Their NuP consisted of their characteristic gelatin-sodium alginate-hyaluronic acid hydrogel containing rat BMSCs and polydopamine (PDA) NPs decorated with TGF-β3, while the AF consisted of the same hydrogel with BMSCs, and PDA NPs decorated with CTGF. Both were printed in a highly specific pattern within a 3D printed PCL scaffold and capped with cartilage endplates made of the same. Both GFs were found to be released steadily over more than 30 days, while good cell viability over this period *in vitro* predicted good differentiation of BMSCs into AF- and NuP-specific tissue *in vivo*. Most notably, these differentiations were observed to be clearly contained within the envisioned NuP/AF divide, with cells in the NuP portion expressing significantly higher GAG/Col II than those in the AF, and cells in the AF expressing higher Col I levels relative to those in the NuP. While the mechanical properties were not ideal (approaching, but not closely matching physiological compressive modulus), this is cause for excitement in this nascent field, showing how *in situ* differentiation along with the spatial control offered by 3D bioprinting can achieve remarkably biomimetic results.

One notable drawback of the cartilage constructs described so far is a phenomenon the authors themselves have highlighted- 3D bioprinted constructs, no matter how biomimetic, tend to elicit an immune response post-implantation that may last for weeks. Several recent works have outlined bioprinting strategies to solve this problem, albeit based more on traditional 3D printing supplemented with later cell seeding rather than direct bioprinting (Sun et al., 2019; 2020a). Zhu et al. (2020) described a PEGDA and decellularized ECM composite cartilage construct loaded with a natural anti-inflammatory agent (honokiol; 3′,5-Di (prop-2-en-1-yl)[1,1′-biphenyl]-2,4′-diol). This was observed to suppress the release of pro-inflammatory cytokines including TNF-α, IL-1β and IL-6 *in vitro* (by approximately 2.5x, 3x, and 2x, respectively, all matching non-inflamed controls except in the case of IL-6). While levels of inflammation were not measured *in vivo* post-implantation, the use of mild natural compounds may certainly be helpful in forming bioprinted constructs that can manage inflammation without stressing the seeded cells. Lee et al. (2019) outlined a possible role for curcumin in bioprinted cartilage constructs (*viz.* a gelatin-curcumin bioink) but did not elaborate on any anti-inflammatory properties observed in their work. Indeed, natural anti-inflammatory, analgesic compounds such as curcumin, gingerol, resveratrol and others hold much promise in this area- Buhrmann et al. (2020) provide a good recent review of these compounds and their myriad benefits in the context of cartilage tissue engineering in particular. Gong et al. (2020) too presented a method wherein anti-inflammatory IL-4 was printed into a GelMA hydrogel/PCL-hydroxyapatite support to achieve lower immune responses in their cartilage regeneration model. *In vivo,* they observed success in the form of higher histological scores and qualitatively better chondrocyte development post-implantation in the IL-4 loaded constructs compared to controls. This incorporation of anti-inflammatories–while not essential–is a relatively under-researched area in the field, and advances in this area will enhance clinical translation immensely. Figure 5 provides a summary and most optimal approach to cartilage bioprinting discussed herein.

## Future Outlook

3D bioprinting is a fast-moving field, and applications of this technology stand to benefit from this rapid pace of advancement. A recent development of interest is Samandari et al. report of multicompartmental bioprinting and its ability to successfully orient cells during printing (Samandari, 2021). The bioprinting of cartilage stands to gain from technologies such as this, as the spatial orientation of chondrocytes in constructs plays a significant role in *in vitro* and -*vivo* success. However, the need for such developments to be complemented by advances in polymer science and more significant contributions from nano- and microscale engineering must be recognized.

There is still much work to be done in the identification of polymer blends that accurately mimic the mechanical properties and biomechanics of natural cartilage, as well as blends that provide high load-bearing and wear-resistant capabilities. As menisci are responsible for stabilizing joints and acting as shock absorbers, mechanical properties remain one of the most important characteristics to consider when designing appropriate implants for meniscal repairs (Inyang and Vaughan, 2020). This is also of particular concern for IVDs, which differ in anatomy and function by location in the spinal column. For instance, in flexion and extension of the lumbar spine, anterior translation of one vertebra or the other should be 8% or less, while posterior translation should be 9% or less. Translation exceeding this is known as alteration of motion segment integrity (AOMSI). Additionally, angular motion of one lumbar vertebra to adjacent vertebra should allow no greater than 15 degrees difference within the first three lumbar units, L1/L2, L2/L3, or L3/L4 or no more than 20 degrees at L4/L5 and 25 degrees at L5/S1 (Keeney, 1984). This is a level of complexity that the field cannot yet achieve but must aim for eventually and must mostly be addressed using polymer science. Further, it has been demonstrated that mechanical properties, as well as mesh size (both of which can be impacted by polymer molecular weight) play a key role in modulating the viability and proliferation of chondrocyte-laden constructs (Lin et al., 2011). In addition, several studies have shown that modifications in polymer surface nanotopography (i.e., surface roughness) can lead to increased chondrocyte density and protein production due to enhanced protein binding on micro- and nano-rough surfaces (Storey and Webster, 2014). Advances in anti-inflammatory polymers or polymeric coatings may be of great use to this field as well-this is an active area of research for surgical implants in general, reviewed recently by both Sánchez-Bodón et al. and Lebaudy et al (Bridges and García, 2008; Al-Khoury et al., 2019; Lebaudy et al., 2021; Sánchez-Bodón et al., 2021)*.* Recent work in this field has also evolved to incorporate decellularized extracellular matrix (dECM) from cartilage and IVD into 3D-printable bioinks to aid in guiding cell proliferation, attachment, and differentiation (Vernengo et al., 2020). For example, Kesti et al. (2015) developed a novel composite bioink comprised of gellan and alginate containing micronized BioCartilage, a commercially available extracellular matrix material native to articular cartilage, which supported chondrocyte proliferation and a favorable deposition of cartilage matrix proteins. While this particular bioink was developed for a broad range of tissue engineering applications, advancements like these may serve as a stepping-stone for the development of more finely-tuned bioinks to better address deficiencies in knee menisci and IVD repair strategies. The factors outlined above must all be considered when developing novel bioink formulations for this area of research.

Slow-release nano- or microformulations encapsulating growth factors and other signaling molecules to encourage proper cell differentiation, or those encapsulating agents such as anti-inflammatories or even anti-microbials to enhance post-implantation success are also of great interest to the field. Novel dual-action molecules tailored for cartilage repair like REG-O3 may be of interest to future researchers in this regard (Montjean et al., 2020). A possible way to integrate all this with traditional bioprinting may be to involve nano- or microscale fibers, such as carbon nanotubes (CNTs), that can lend structural support to constructs as well as releasing such agents from their core or their surface. Szymański (2020) provide an excellent review of recent tissue engineering work that has involved CNTs. Capable of self-assembly into nanostructures and acting as cell adhesion points or growth factor binding sites, peptide amphiphiles are also possible additives to cartilage bioinks that are already being explored in other tissue engineering approaches (Lewis et al., 2020; Di Marzio et al., 2020). The next wave of bioink development should also most certainly focus on biomaterials that exhibit 4D bioprinting properties, consisting of materials that can be 3D printed, yet also respond to environmental stimuli (e.g., temperature, pH, etc.) over a desired duration of time (Saska et al., 2021). For example, Betsch et al. (2018) developed a magnetically responsive iron nanoparticle-based bioink capable of producing constructs exhibiting alternating layers of aligned and random collagen fibers able to more accurately recapitulate the complex architecture of native articular cartilage, leading to enhanced collagen II production. Multilayered nano-/microparticles or -fibers can lend additional complexity to cartilage bioprinting by effecting fine control of chondrocyte differentiation and growth through temporally-controlled release of growth factors and other signaling molecules (Go et al., 2011; Asiri et al., 2021). Despite the availability of a considerable body of non-human *in vivo* data, 3D bioprinted cartilage constructs have–so far–not moved on to clinical trials. While fundamental research paves the way to more advanced applications, a field as nascent as this will eventually require clinical data for the refinement and evolution of the field, identifying shortcomings that exist in the *in vitro* development process that impact downstream clinical translation. In this light, it is heartening to note that such trials are now almost underway, with 3DBio Therapeutics’ AuriNovo technology (3D bioprinted auricular cartilage for microtia) on the verge of recruiting trial subjects (NCT04399239). The technology is based on a proprietary bioink containing patient-derived (autologous) chondrocytes (AuriNovo for Auricular Reconstruction, 2020). This technology has been spurred in part by the introduction of personalized medicine to this field-a development rooted in the problem of possible implant rejection in a clinical setting as discussed earlier, and the autologous approaches needed to overcome it (Edri et al., 2019).

The need for printed constructs to encourage proper differentiation of seeded stem cells in the correct spatial orientation and patterning is another area that merits further investigation. The majority of works discussed in this review have opted for a static pore size, for instance. While there is evidence in literature that pore sizes of 100–300 μm are optimal for cell differentiation and orientation within constructs, anisotropy in both this and in the release of growth factors, anti-inflammatory agents, anti-microbials etc. from nano/microparticles or -fibers may provide exciting new avenues of producing constructs that are more physiologically relevant, particularly when combined. This is highlighted by Sun and others’ work (Zhang et al., 2014; Han et al., 2015; Naseri et al., 2016; Song et al., 2017; Sun et al., 2020a).

As tissue constructs go, cartilage has a relatively simple and straightforward structure to attempt to mimic than many other tissues in the human body. A successful convergence of new and improved polymers, precise bioprinting techniques focusing on anisotropy and dedicated “regions” of cartilage structure, optimized additives, and translation-friendly cell isolation, all complemented with regular feedback in the form of clinical data will define the direction this promising technology will take in the coming years.
